# Role of CT scan in diagnosis of renal lymphangiectasia: our single-center experience

**DOI:** 10.1080/0886022X.2017.1337584

**Published:** 2017-06-22

**Authors:** Vaidehi K. Pandya, Harsh C. Sutariya, Shruti P. Gandhi, Sajni I. Khemchandani, Himanshu V. Patel, Maulin K. Shah

**Affiliations:** a Department of Radio Diagnosis and Imaging., G.R. Doshi and K.M. Mehta Institute of Kidney Diseases and Research Centre (IKDRC) and Dr. H.L. Trivedi Institute of Transplantation Sciences (ITS), Civil Hospital Campus, Asarwa, Ahmedabad, Gujarat, India;; b Department of Transplantation surgery and Urology, G.R. Doshi and K.M. Mehta Institute of Kidney Diseases and Research Centre (IKDRC) and Dr. H.L. Trivedi Institute of Transplantation Sciences (ITS), Civil Hospital Campus, Asarwa, Ahmedabad, Gujarat, India;; c Department of Nephrology, G.R. Doshi and K.M. Mehta Institute of Kidney Diseases and Research Centre (IKDRC) and Dr. H.L. Trivedi Institute of Transplantation Sciences (ITS), Civil Hospital Campus, Asarwa, Ahmedabad, Gujarat, India

**Keywords:** Renal lymphangiectasia, CT scan, lymphatic malformation

## Abstract

**Background:** Renal lymphangiectasia is rarely reported benign renal disorder of lymphatic malformation. Though found incidentally; it presents with nonspecific symptoms and shows characteristic findings in radiological imaging studies.

**Aim:** Here, we report eight patients with symptoms, laboratory and imaging findings compatible with renal lymphangiectasia. This report describes clinical and laboratory characteristics, treatment, Imaging findings and outcome of a series of patients with renal lymphangiectasia and reviews the literature.

**Methods and material:** Eight patients (mean age 45 years, male:female ratio 3:1) from 1st January 2011 to 30th June 2016; showing renal lymphangiectasia as incidental finding on CT IVP were included in the series. Imaging and laboratory findings were reviewed. Two out of eight patients (25%) underwent aspiration of collection and laboratory findings confirmed the diagnosis of renal lymphangiectasia. Four out of eight patients (50%) did not undergo aspiration of fluid and were offered conservative treatment. Two out of eight patients (25%) were donors for renal transplantation who were managed conservatively.

**Results:** Renal lymphangiectasia was diagnosed on CT IVP. In each case, where aspiration of collection fluid was offered, the laboratory diagnosis of renal lymphangiectasia was confirmed and patients were managed conservatively. However, large collection in one patient was relieved by percutaneous aspiration.

**Conclusions:** Renal lymphangiectasia can be diagnosed with CT scan and confirmed by laboratory tests. As it may be confused with other cystic lesions of kidney; proper diagnosis and exclusion of other differentials can be effectively offered by CT scan IVP, which can avoid unnecessary invasive treatment options.

## Introduction

Renal lymphangiectasia is very rarely reported benign condition of lymphatic malformation where dilated perirenal, peripelvic or intrarenal lymphatics are seen [1,2]. The main causative factor is noncommunication of perirenal and peripelvic lymphatic channels with the main lymphatics [3]. It is observed both in adults and in children of both sexes [3,4]. It may be unilateral or bilateral [5–7]. Various causes identified are familial, developmental and acquired causes. Mostly, asymptomatic, it may show nonspecific symptoms and is diagnosed by typical imaging findings.

The condition is benign but is commonly misdiagnosed as other cystic renal diseases and hydronephrosis [8]. Hence, its thorough knowledge and typical imaging appearances will help the radiologist to diagnose it and offers appropriate management of the patient.

Most of the information about this entity comes from isolated case reports. There are scarce reports of multiple cases reported as case study with comparison. Here, we report a case study of eight patients with comparison in the context of presenting symptoms, imaging findings, treatment options and laboratory confirmation where possible.

## Materials and method

We retrogradely searched about 9900 patients who underwent contrast-enhanced CT scan at Institute of Kidney Disease and Research Center, Dr. H.L. Trivedi Institute of transplantation sciences, Asarwa, Ahmedabad between 1st January 2011 to 30th June 2016 and found eight patients diagnosed to have renal lymphangiectasia as an incidental finding. CT scan was done on Seimens Somatom 64 Slices CT scanner with Injection of 350 mL iohexol in 60 mg/kg dose with prior written consent. Per cutaneous aspiration of the collection was offered where the location was favorable and laboratory investigations were done for fluid cytology, culture, glucose, protein and triglyceride levels. According to the laboratory investigations, diagnosis of renal lymphangiectasia was confirmed and the patients were managed conservatively. Two out of eight patients (25%) underwent aspiration of collection and laboratory findings confirmed the diagnosis of renal lymphangiectasia. Four out of eight patients (50%) did not undergo aspiration of fluid due to unfavorable site of collections so they were offered conservative treatment. Two out of eight patients (25%) were donors for renal transplantation and they were managed conservatively. Only one of the eight patients who had large collection causing compression symptoms underwent percutaneous drainage of collection, and on follow–up, total resolvement of renal lymphangiectasia collection was achieved.

## Results

**Figure 1.**
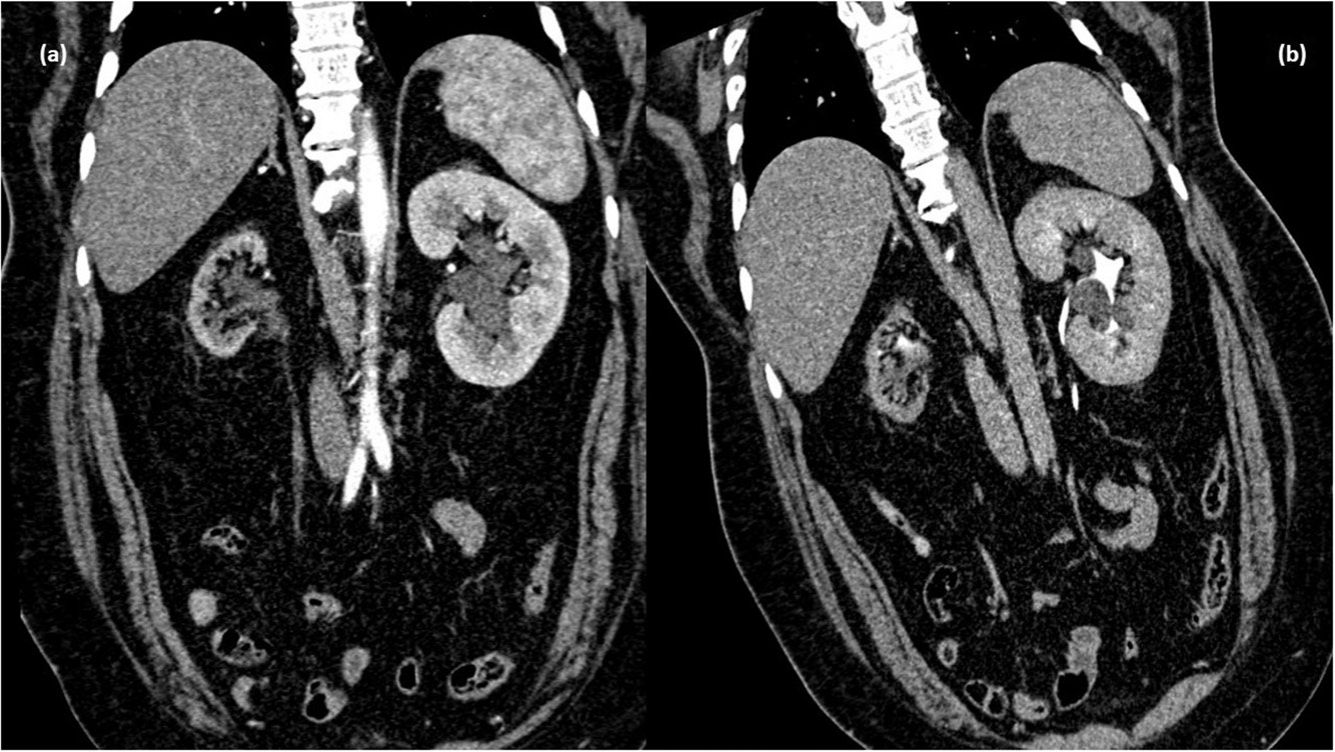
CT scan arterial phase in coronal plane showing non enhancing collection in peripelvic region of left kidney (d) MPR reformation of coronal plane of delayed phase of CT scan abdomen showing normal excretion of contrast with no evidence of leakage in PC system – unilateral peripelvic lymphangiectasia.

**Figure 2.**
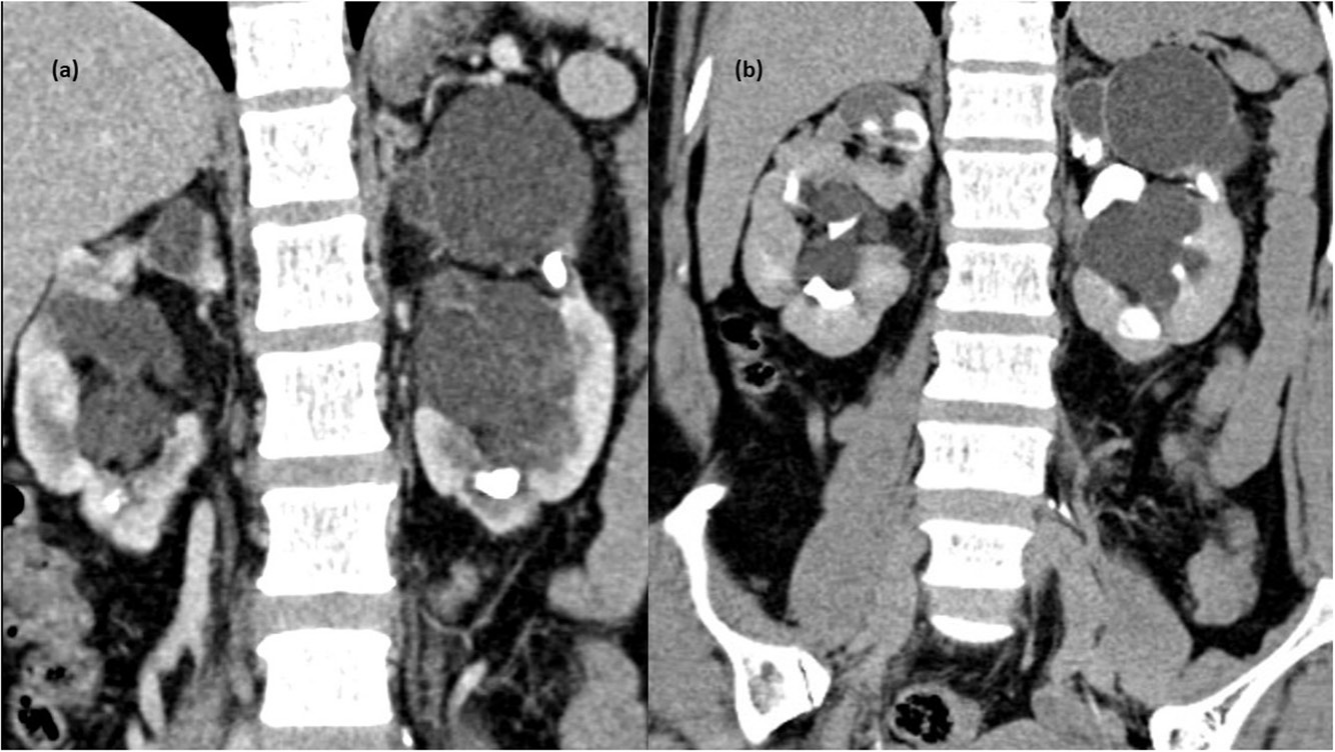
CT scan venous phase in coronal plane showing nonenhancing collection in peripelvic region of both kidneys (d) MPR reformation of coronal plane of delayed phase of CT scan abdomen showing normal excretion of contrast with no evidence of leakage in PC system. Bilateral peripelvic lymphangiectasia. Associated cystic lesions are also seen.

**Figure 3.**
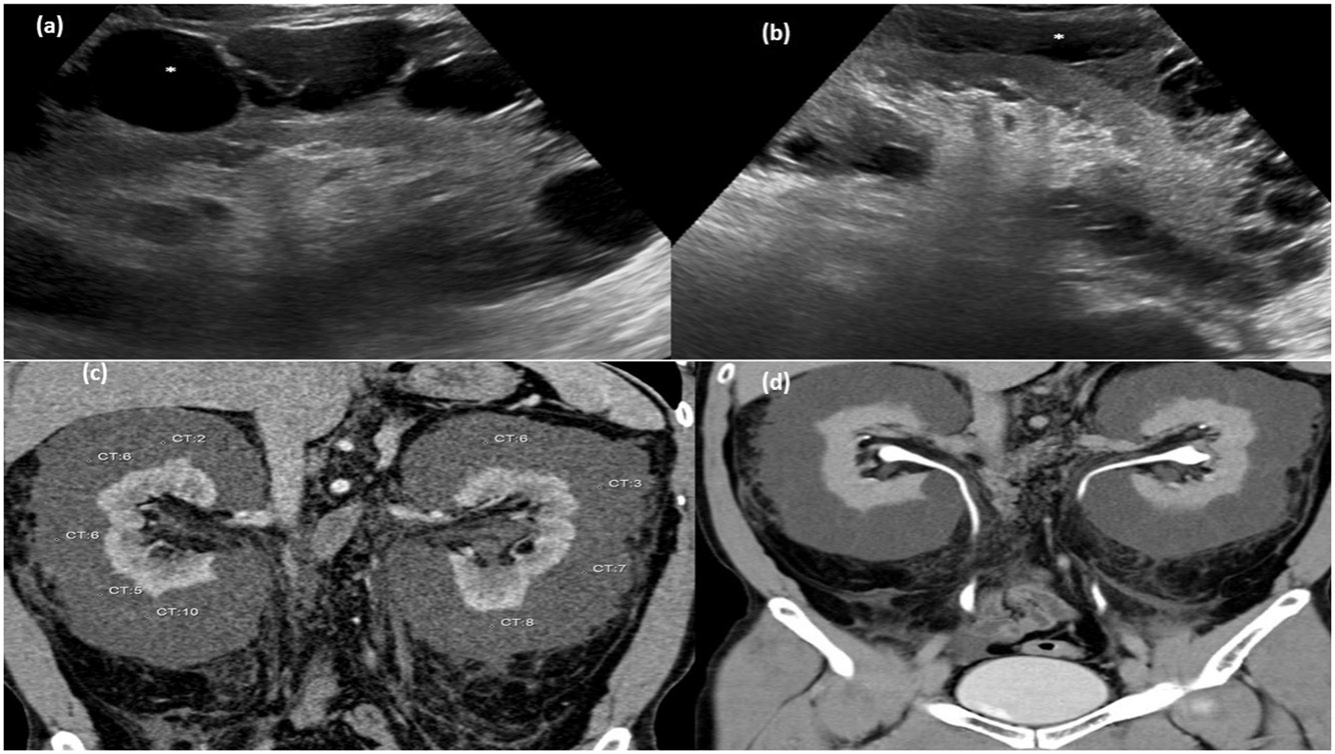
Gray-scale ultrasound image of right kidney (a) and left kidney (b) showing multiseptated anechoic collection (asterisk) in perinephric region. (c) CT scan arterial phase in coronal plane showing multiseptated nonenhancing collection with average HU density 0–10 HU. (d) MPR reformation of coronal plane of delayed phase of CT scan abdomen showing normal excretion of contrast.

**Figure 4.**
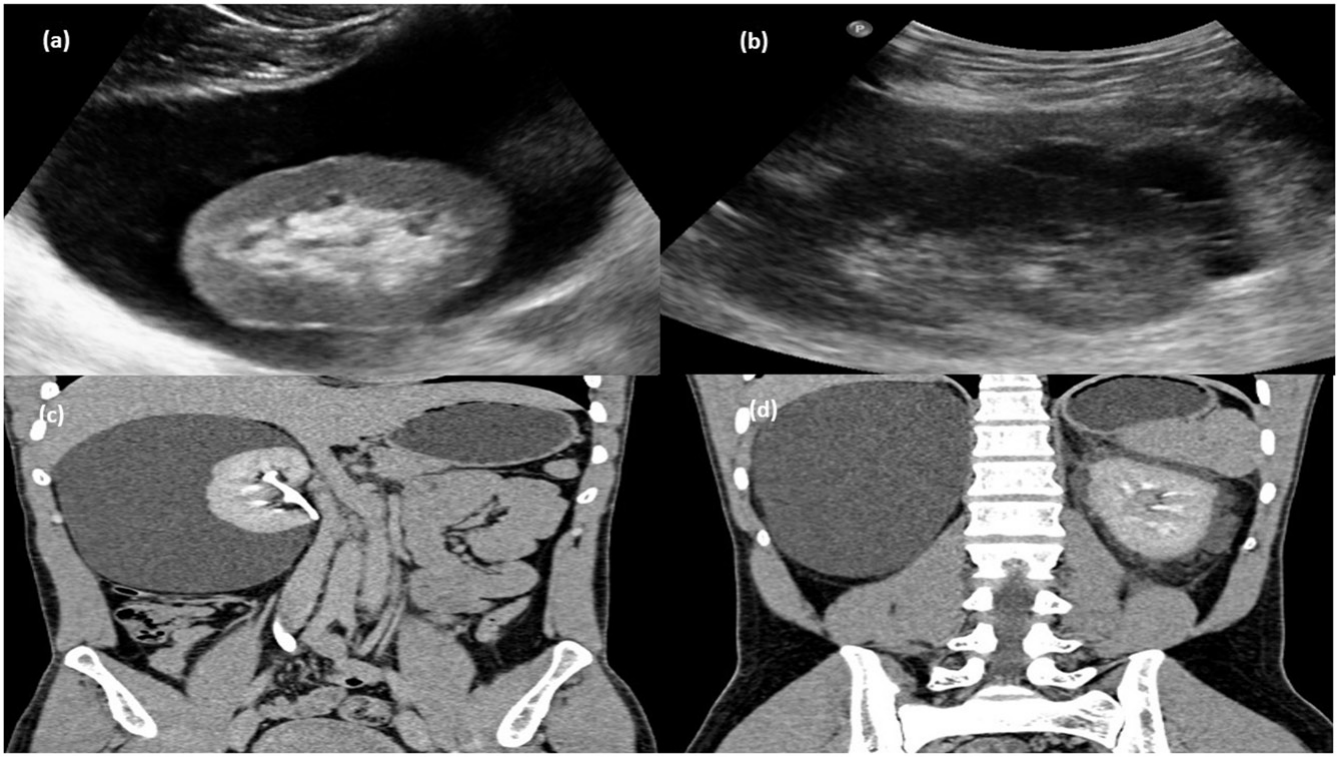
Gray-scale ultrasound image (a) of right kidney showing anechoiec collection in perinephric region and (b) left kidney showing multi septated anechoic collection in perinephric region. (c) CT scan arterial phase in coronal plane showing nonenhancing collection (d) MPR reformation of coronal plane of delayed phase of CT scan abdomen showing normal excretion of contrast.

**Figure 5.**
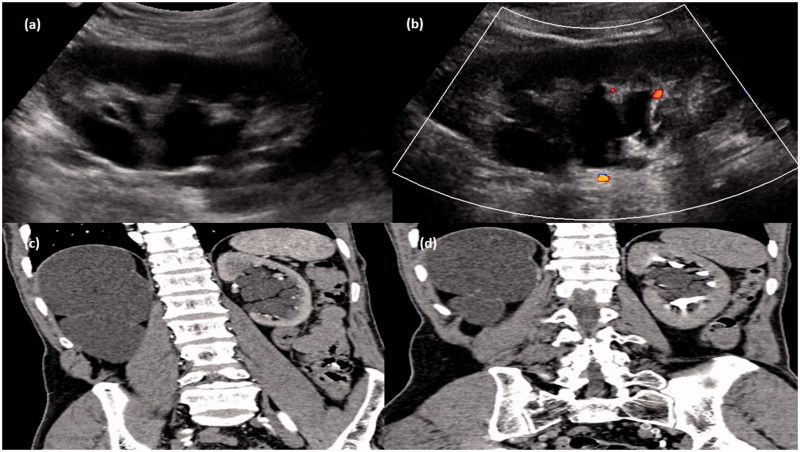
Gray-scale ultrasound image (a) of left kidney showing an echoiec collection in peripelvic region which showed no evidence of color flow (b) on color Doppler study. CT scan arterial phase (c) and delayed phase (d) in coronal plane showing collection in peripelvic region with no evidence of contrast within it. The grossly hydronephrotic contralateral kidney is also seen.

About 9900 patients of all ages and both sexes, undergoing contrast-enhanced CT scan were searched retrogradely for evidence of renal lymphangiectasia. Age of patients diagnosed with renal lymphangiectasia was mean 45 years (28–62 years) with male-to-female ratio 3:1 (male:female). Five patients out of eight patients (62.5%) presented with complain of flank pain. None of them had complain of hematuria. Only one patient had associated hypertension. The demographical criteria of patients with presenting symptoms and the presence of comorbidity are described in Table 1. The collection of two out of eight patients (25%) were drained percutaneously and the diagnosis of renal lymphangiectasia was confirmed on laboratory tests (Table 2). Four of them (50%) could not undergo drainage of the collection due to para pelvic location of collection. They were managed conservatively; one of these patients had associated prostatic enlargement and received treatment accordingly. Two of eight patients were healthy adults who were being evaluated for live related kidney donation. They also had para pelvic renal lymphangiectasia and did not undergo drainage. One of them had bilateral para pelvic lymphangiectasia (Figure 1) and other had unilateral para pelvic lymphangiectasia (Figure 2); both of them were not taken as donors. Out of eight patients, six (75%) had peripelvic lymphangiectasia, and only two (25%) had perinephric lymphangiectasia. Four (50%) patients out of them had bilateral disease and rest four (50%) of them had unilateral disease. Though it is not documented in any literature, we observed that if the disease is unilateral, it involves left side more frequently (Table 2). On primary ultrasound evaluation outside or at our center, One of the eight patients (12.5%) was diagnosed as polycystic kidneys (Figure 3), one of them (12.5%) was diagnosed to have urinoma (Figure 4) and rest of them (75%) were reported as hydronephrosis (Figure 5) on ultrasound. Only two of the eight patients were offered aspiration of collection and laboratory investigation of aspirated fluid. In both the patients, laboratory tests favored diagnosis of renal lymphangiectasia, as they showed the presence of majority of lymphocytes (80–90%), increased protein and fat levels and negative test for culture and sensitivity (Table 3). All the patients were properly diagnosed on CT scan IVP and other differential diagnosis were excluded. Prompt diagnosis on CT IVP could assist the treating doctor in further management and to avoid unnecessary invasive procedures. Diagnosis on CT scan IVP could help the transplant surgeon to decide the proper donor and helped the patient as well as the donor to prevent unwanted complications.

**Table 1.** Demographical criteria of patients with presenting symptoms and the presence of comorbidity.

			Symptoms		
Sr. No	Age (in yrs)	sex	Flank pain	Hematuria	Comorbidity
1	34	M	NO	NO	NO
2	28	M	YES	NO	NO
3	50	M	YES	NO	NO
4	40	F	YES	NO	YES (Hypertension)
5	74	M	YES	NO	NO
6	45	M	YES	NO	NO
7	51	M	NO	NO	NO
8	62	F	NO	NO	NO

**Table 2.** Diagnosis on ultrasound and CT scan with type of involvement.

Sr. No	Diagnosis on USG	Type of lymphangiectasia	Involvement	Aspiration done
1	Poly cystic kidneys	Perinephric	Bilateral	Yes
2	Urinoma	Perinephric	Bilateral (R > L)	Yes
3	PUJ obstruction	Peripelvic	Unilateral (Left)	No
4	Hydronephrosis	Peripelvic	Bilateral	No
5	Hydronephrosis	Peripelvic	Unilateral (Left)	No
6	Hydronephrosis	Peripelvic	Unilateral (Left)	No
7	Hydronephrosis	Peripelvic	Unilateral (Left)	No
8	Hydronephrosis	Peripelvic	Bilateral	No

**Table 3.** Comparison of laboratory investigations of aspirated fluid.

Sr no.	Age	Sex	Primary diagnosis	Leucocyte count	Protein level	Triglyceride	Glucose	Culture and sensitivity
1	34 yr	M	Poly cystic kidneys	55/cmm	550 mg/dl	increased	normal	negative
2	28 yr	M	Urinoma	scattered	565 mg/dl	normal	normal	negative

## Discussion

Renal lymphangiectasia is also known as renal lymphangiomatosis, hygroma renale and polycystic disease of the renal sinus [1,9,10]. It is very rare benign lymphatic malformation of renal lymphatics. The exact pathogenesis is unclear. However, congenital occurrence is thought of as there are familial associations of the disease. Familial predilection is seen in very few reported cases [1]. Only Meredith et al. have supported the familial nature of the disease, having found exacerbation of renal lymphangiomatosis during pregnancy in two sisters [3]. However, no family association was found in any of the patients included in this study. Another proposed theory is for an acquired cause, which suggests that the lymphatic vessels may get blocked due to inflammation or other obstruction and causes lymphangiectasia [11]. Finally, some of them also proposed the lesions to be true neoplasm [12].

Usually, the renal lymphatic ducts drain in to larger retroperitoneal lymphatics. Failure of such drainage leads to dilatation of these ducts and formation of unilocular or multilocular collections in perinephric spaces or pelvic sinuses [10]. Two patterns of cystic lesions in renal sinus are identified. When multiple confluent small cysts are seen in renal sinus, they are intraparenchymal, peripelvic and usually bilateral and benign. Another pattern defines large cyst inside the renal sinus that originates in medial renal parenchyma and encroaches in to renal sinus. These cysts may be single or few in number para pelvic cysts, which appears same as cortical cysts on imaging. When the radiological and histological findings are not correlated, these lesions are broadly classified as cystic lesions of renal sinus [13]. We found about 25% patients to have perinephric lymphangiectasia and about 75% patients of peripelvic lymphangiectasia. However, no data in literature for individual incidence of perinephric or peripelvic lymphangiectasia were found.

The clinical presentation of this condition is quite variable. Commonly, it is asymptomatic and diagnosed incidentally. However, it may show sudden appearance and rapid growth or cessation of growth and spontaneous regression of symptoms [12]. However, when present the symptoms may be flank pain, abdominal mass, hematuria, ascites, lower extremity edema, hypertension and renal failure [10]. Renal function is usually not impaired. The presence of hypertension is presumed to be due to subcapsular collection causing compression of renal parenchyma and resulting in excessive renin release.

In asymptomatic cases also, imaging studies give a striking clue to the diagnosis. Ultrasound studies reveal multiseptated thin-walled fluid collections in perinephric or peripelvic regions with normal or enlarged kidneys [6]. Renal cortical echo texture may be normal or increased with preserved or lost corticomedullary differentiation. It may also present as a solid mass when the small intra renal lymphatics are obstructed [14]. Ascites may also be seen on ultrasound. On CT scan, RLM appears as a well-defined low attenuation (0–20 HU density) multiseptated collections in perinephric or peripelvic regions with normal renal parenchymal enhancement and contrast excretion. Higher density fluid may represent intra cystic hemorrhage. No evidence of invasion of other organs is seen by such collection. The presence of fluid or fluid-filled lesions in the retro-peritoneum around great vessels and crossing the midline at the level of origin of renal vessels is typical CT sign of RLM [4]. These are the dilated renal lymphatics, which are draining in to larger retroperitoneal lymphatics.

The confirmation of diagnosis is done by percutaneous fluid aspiration and cytology of aspirated fluid. The fluid in RLM appears to be sterile, chylous fluid containing majority of lymphocytes (more than 90%), small amount of fat and protein. High levels of renin in the aspirated fluid confirms the origin to be renal. However, due to unavailability, we have not correlated renin levels in any of the patients included in our study.

The contents of renal lymphatic aspirates are different from that of thoracic duct because it is outside the mesenteric drainage pathway [10,15].

Most common differential diagnosis are polycystic kidneys, urinoma and hydronephrosis [9,10,16]. In polycystic kidneys, the renal cortex shows the presence of cysts distorting renal parenchyma and causing splaying of PC system. Whereas in RLM, the renal cortex appears normal. Similarly, in RLM, the PC system appears normal in contrast to hydronephrosis. Urinoma also presents as cystic collection in perinephric region. However, it may be associated with the evidence of obstruction in PC system and may show leakage of contrast medium in delayed phase of CT IVP to indicate connection of collection with PC system. Other renal cystic masses like nephroblastomatosis, lymphoma, multilocular cystic nephroma are also included in differentials. They can be differentiated from RLM on CT scan as all of them involve the renal parenchyma. Nephroblastomatosis and lymphoma are solid in attenuation in contrast to fluid attenuation of RLM [2,9].

Perirenal liposarcoma shows the presence of macroscopic fat [16].

Untreated cases can be complicated by hematuria, ascites, hypertension, infection, renal vein thrombosis or altered renal function [9,17]. Usually, asymptomatic cases do not require treatment. Pain on account of compression due to the presence of collection is usually relieved by percutaneous aspiration of fluid. However, hypertension and altered renal function improve after conservative treatment [4,9]. Asymptomatic patients receive conservative management, but symptomatic large collections are treated by mar-supialization or nephrostomy [9]. Antihypertensive drugs can be used in hypertensive patients for symptomatic relief. Page kidney and renal vein thrombosis are one of the rare complications where nephrectomy is done as a treatment [9]. However, nephrectomy is not considered a choice nowadays, as in case of asymmetrical bilateral involvement, the cysts in the contralateral kidney may increase in size [7,18]. Antibiotic treatment is given only in cases of secondary infection in the lesions [19] and may decrease the cystic lesions and perirenal fluid accumulation, which is associated with concomitant urinary tract infection. Here, in our study, seven out of eight patients received conservative treatment. Out of two patients who underwent per cutaneous aspiration, one has shown total resolvement in the collection and associated symptoms while as the other patient underwent only diagnostic aspiration and was managed conservatively. All the patients are doing well on follow-up.

## Conclusions

Renal lymphangiectasia is a very rarely reported benign, usually asymptomatic condition where imaging studies can play a very important role in differentiation from other conditions. During the course of the disease, the size of dilated lymphatic vessels may increase, obstruct the pelvicalyceal system and may cause chronic pyelonephritis and hypertension later. Early and previse diagnosis by means of CT scan can guide the proper and timely management. Imaging studies also help in determining the extension of the fluid collection and reduces the morbidity associated with it. In difficult cases, aspiration of fluid can confirm the diagnosis and can avoid the unnecessary surgical interventions with their associated complications. Awareness about this condition will result in early diagnosis, early treatment and reduced morbidity.
